# A national survey on community pharmacists’ perception, practice and perceived barriers towards pharmaceutical care services in the United Arab Emirates

**DOI:** 10.1080/20523211.2025.2523936

**Published:** 2025-07-08

**Authors:** Salma Mohamed, Subish Palaian, Muaed Alomar, Mohammad M. Al-Ahmad

**Affiliations:** a Department of Clinical Sciences, College of Pharmacy and Health Sciences, Ajman University, Ajman, UAE; b Department of Clinical Pharmacy, College of Pharmacy, Al Ain University, Al Ain, UAE

**Keywords:** Pharmaceutical care, community pharmacies, community pharmacist, perception, practice, barriers, United Arab Emirates

## Abstract

**Background:**

Pharmaceutical care (PC) is less practised in United Arab Emirates (UAE) community pharmacies. This study assessed community pharmacists’ (CPs) perceptions, practices and perceived barriers to providing PC.

**Methods:**

A nationwide cross-sectional survey was conducted from May to October 2024 among CPs through direct visits, email invitations and WhatsApp groups. Customised version of a previously validated 5-point Likert-type questionnaire was used (Cronbach alpha = 0.93). Individual statements were scored 1–5, and Mann Whitney and Kruskal Wallis tests were performed to find the association between total perception, practice and barrier scores with demographic variables. Post hoc analyses and Kendall’s correlation were performed wherever applicable, at alpha = 0.05.

**Results:**

A total of 227 CPs, with 70.9% (*n* = 161) of bachelor in pharmacy degree holders responded. The total median (IQR) scores for perception, practice and barriers were 24 (22–26)/30, 40 (34–45)/50 and 76 (63–86)/125, respectively. Most of the CPs felt that patients’ medications should be reviewed by them to prevent medicine-related errors and promote the appropriate medication use [median (IQR) 5 (4–5)]. They also felt that CPs are professionally skilled in providing PC [median (IQR) 5 (4–5)]. The major barrier reported was the lack of support from other health professionals toward PC [median (IQR) 4 (3–5)]. There was a statistically significant association between total perception scores with age (*p* = 0.023), work experience (0.036) and working hours (*p* = 0.012), total practice scores with work experience (*p* = 0.035) and training in PC (*p* = 0.009) and total barrier scores with the average number of CPs available in the shift (*p* = 0.002). A significant correlation was noticed within a few perception, practice and barrier constructs and between these constructs and participants’ demographic characteristics, *p* < 0.05.

**Conclusion:**

Specific interventions at academic and regulatory levels targeting specific barriers are urgently needed with the incorporation of patient-centred care and interprofessional collaboration in academic and practice settings as the starting point.

## Background

Over the last four decades, the role of pharmacists has undergone significant changes and pharmacists have shifted from simply compounding and dispensing medications to offering a wide range of patient care services. After the definition of pharmaceutical care (PC) by Hepler and Strand as ‘a responsible provision of drug therapy to achieve definite outcomes that improve a patient’s quality of life’ (Hepler & Strand, 1990), the concept of PC is practised widely shifting its primary focus from plain medication supply to a patient-centred approach that highlights individualised drug therapy (Tang et al., 2020). The main components of PC are patient assessment, medication management, individualised care plan followed by follow-up evaluation. Motivated by this turn, pharmacists started to give priority to patient-centred practice rather than traditional dispensing practice (Mishore et al., 2020).

The philosophy of PC has been promoted, since 1990, and its effectiveness is well defined throughout the research. The PC is currently provided globally and benefits patients, doctors and pharmacists all at the same time (Kopciuch et al., 2021). On the ground, many barriers are hindering PC practice including the economic structure of retail pharmacies (Tang et al., 2020), the lack of synergy between professions and the inappropriate design of the pharmacy (Siddique et al., 2022).

Considered an essential part of the healthcare team, community pharmacists (CPs) have proven their valuable role over time. Reducing medication-related errors, improving medical outcomes and enhancing the patient quality of life were the main advantages of their interventions. The effectiveness of patient-centred care relies on CPs' skills and knowledge as well as their collaboration with other healthcare professionals can provide the best care for the patients (Jarab et al., 2024).

In UAE, PC is now a central course or as part of study curricula by most pharmacy colleges. The BPharm programmes are offered by eight pharmacy colleges and the Doctor of Pharmacy (PharmD) by one college pointed out on PC (Ashames, 2019). At the time of the study, there were 11,153 CPs in the UAE serving a population of 10 million, according to the UAE statistical report from the Ministry of Health in 2020 (MOHAP, 2022). The place of CPs in society is still unclear and undervalued. Corresponding reasons had been studied by authors of a 2014 study on pharmacists’ roles in the United Arab Emirates as they identified several difficulties and obstacles to maximising pharmacy services (Kharaba et al., 2022).

Studies in the UAE were limited to/focused on either a single city, as shown in the study of Rayes et al. (2015) in Dubai, or have examined both hospital and community environments, such as the work done by Kharaba et al. (2022). Other studies, such as the one illustrated by Tawfiq et al. (2021), focused on pharmacy students, while some, like that of Ghazal (2014), examined specific challenges like barriers to practice. Furthermore, the topic of PC has been studied among GCC countries by different researchers who also had the same focus as the UAE. For example, either on a single city such as Siddique et al. (2022) study conducted in Saudi Arabia, a hospital and community-focused, such as El Hajj et al. (2016) research conducted in Qatar or a hospital focused, such as Katoue et al. (2014) and Awad et al. (2006) conducted in Kuwait. A broad overlook of the studies from GCC showed a few specific studies on CPs' perceptions, practices and barriers towards PC. The same observation is noted from MENA studies. For instance, in Jarab et al. (2024) study conducted in Jordan, the focus was on the hospital setting while in the Iranian study conducted by Mehralian et al. (2014) team, the area of the study was narrowed to barriers of PC. Sallom et al. (2023) evaluated PC studies in the MENA region and concluded that the a high need for more comprehensive studies in the PC field.

As UAE is one of the countries in the GCC, the findings of this study can be generalised and an important benchmarking for future studies in the region can be drawn. Hence, the present study was aimed at assessing the perception level and practice towards the provision of PC and determining the major barriers faced against the implementation of PC.

## Methods

### Study design

In this cross-sectional study, questionnaire-based survey, the research questionnaire was distributed among CPs with a variety of education, experience and qualifications. It was distributed to pharmacists who work in community pharmacies either in independent or chain pharmacies.

### Study setting

The study was conducted in community pharmacies throughout UAE covering all seven emirates, including Abu Dhabi, Dubai, Sharjah, Ajman, Umm Al-Quwain, Ras Al Khaimah and Fujairah.

### Study timeline

The study was conducted from May 2024 to October 2024. The timeline was divided into the preparation phase, the data collection phase followed by the analysis phase.

### Ethical approval and informed consent

The ethical approval was obtained from the Ethical Review Board of Ajman University, on May 16th, 2023, (Ref Number: P-F-F-16-May-i). The agreement of participants was confirmed before collecting the responses and that was considered as written consent. All methods applied were carried out in compliance with the appropriate guidelines and protocol by the ethical approval body.

### Study subjects

The sample for this study comprised pharmacists working in community pharmacies across all seven emirates of the United Arab Emirates. The study included pharmacists of all nationalities from both genders and required the participants to have a working understanding of the English language.

### Inclusion and exclusion criteria

This study was intended only for CPs who work in community pharmacies in UAE. The primary inclusion criteria were being a licened CP in UAE regardless of years of experience. Assistant pharmacists and trainees were excluded as they had different responsibilities, education and training.

### Sample size

The sample size was calculated using the Raosoft sample size calculator with a confidence interval of 95% and a margin of error of 5% (Raosoft, 2023). A sample size of 372 was calculated by referring to the total number of licensed pharmacists working in UAE which was around 11,153 as per the last update at the time of the study (MOHAP, 2022).

### Sampling method

In this research, a stratified sampling method was followed to ensure that the sample would be representative and to reduce sampling bias and improve generalisability. The representative samples from each emirate have been calculated based on the total number of pharmacists working in community pharmacies.

### Questionnaire

The English version of the questionnaire used was obtained from Shrestha et al. (2022). The same questionnaire was used with small modifications needed in order to be applied in community pharmacies. One question was changed to be suitable for the community situation (current site of work changed to community pharmacy: chain or independent). Two questions had been removed. The first question is about the services provision of the pharmacy: outpatient, inpatient or both and the second question is about the bed size of the associated hospital, as they are not applicable to the CP situation. The final questionnaire consisted of five segments: pharmacist demographics and work-related questions, perception-related questions, practice-related questions, drug therapy-related problems (DTRPs) related questions and barrier-related questions with 11, 6, 10, 4 and 25 questions, respectively. The total number of questions was 56 (Appendix 1). The questionnaire was scored on the five-point Likert scale (1 = strongly disagree to 5 = strongly agree) for the perception and barriers sections. For the practice section, the scale was from 1 = never to 5 = all the time. Questions of demographic data and DTRPs were multiple choice questions.

### Data collection

The online survey link for data collection was shared through direct visits of the researcher to the pharmacy, email, and WhatsApp groups. The author visited a total of 175 pharmacies across the UAE. During these visits, the purpose of the study was explained to the pharmacist who was invited to click on the survey link to participate and considered this as a consent agreement. For pharmacies that could not be visited in person, the survey link was sent to the official email along with the detailed explanation or shared through WhatsApp groups of pharmacists in UAE which is commonly used for communication and networking. The first page of the survey contained the objective, nature, benefit of the study and researcher contact details. Filling the questionnaire was considered as written consent (mentioned on the first page of the questionnaire).

The data were collected across all emirates, including remote emirates, such as Ras Al Khaimah and Fujairah, to ensure that the findings are representative and to enhance the validity and applicability of the findings.

### Pilot testing

A pilot study was conducted on 10 CPs to check for the reliability of the questionnaire. The reliability of the tool was estimated based on Cronbach’s Alpha (alpha = 0.935). Since the Cronbach alpha score was high and no changes were needed after reliability testing, the responses of the pilot study were involved in the main survey.

### Data analysis

The Statistical Package for Social Sciences (SPSS) version 30. Was used for the entry and analysis of the collected data. To summarise demographic information and responses to multiple-choice questions, descriptive statistics were used. Descriptive statistics (i.e. mean, frequency and percentage) and inferential statistics (Kendall’s correlation) were applied as suitable. Kendall’s tau values were translated as less than ±0.25 (very weak), ±0.25 to ±0.34 (weak), ±0.35 to ±0.39 (moderate) and ±0.40 or larger (strong relationship). To compare perception, practice and barriers with other variables, the Mann–Whitney U test was used for dichotomous variables and the Kruskal Wallis test for 3 or more other variables at alpha is equal to 0.05. When *p* value <0.05 it was considered statistically significant at a 95% confidence interval. Post hoc (Bonferroni test) analyses were performed for statistically significant variables with more than two subgroups.

## Results

### Participants’ distribution across the seven Emirates

A total of 227 PCs had participated with a response rate of 61%. The highest percentages of pharmacists were from Sharjah (30.39%) and Abu Dhabi (29.51%), while Ras Al Khaimah, Fujairah and Umm Al Quwain, have significantly lower representation by 3.52%, 2.64% and 2.20%, respectively. A notable distribution that showed different Emirates’ rates of participation is available in Table 1.

**Table 1.** Distribution of participating pharmacists among the seven Emirates.

Emirates of UAE	The total no of participants from each Emirate	
	Number	Percentage (%)
Sharjah	69	30.39
Abu Dhabi	67	29.51
Ajman	38	16.74
Dubai	34	14.97
Ras al Khaimah	8	3.52
Fujairah	6	2.64
Umm al Quwain	5	2.20
Total	**227**	100

### Demographic distribution among participants community pharmacists

The total respondents’ number was 227 PCs. Table 2 provides valuable information on the demographics, qualifications, experience, and work environments of participant pharmacists in the study. The majority of participants were young professionals (22–31 years), with a higher representation of females (68.3%) among the pharmacists surveyed. The local differences also draw attention to less represented Emirates like Umm Al Quwain, and Fujairah by (2.2% and 2.6%, respectively) vs the highest represented like Sharjah (68%). Most pharmacists were employed full-time by chain pharmacies (79.7%). The majority of them work with between 11 and 110 prescriptions a day (60.8%). Most participants were holders of a Bachelor of Pharmacy (BPharm) degree (70.9%), with a considerable proportion of participants (76.2%) having less than 10 years of experience. Other details will be available in Table 2.

**Table 2.** Demographic characteristics of study participants.

Study variables	Frequency (%)
Age (in years)	
≤21	9 (4)
22–31	146 (64.3)
32–41	66 (29.1)
52+	6 (2.6)
Gender	
Male	72 (31.7)
Female	155 (68.3)
Qualification	
MPharm	31 (13.7)
PharmD	35 (15.4)
BPharm	161 (70.9)
Work experiences (in years)	
≤1	17 (7.5)
1–10	173 (76.2)
10.1–20.0	34 (15)
20.1–30.0	0
30.1+	3 (1.3)
Training in pharmaceutical care	
Received	180 (79.3)
Not received	47 (20.7)
The current site of work	
Community pharmacy (chain pharmacy)	181 (79.7)
Community pharmacy (independent pharmacy)	46 (20.3)
Working hours per week	
≤34	45 (19.8)
35+	182 (80.2)
Daily number of prescriptions handled	
≤10	68 (30)
11–110	138 (60.8)
111–210	12 (5.3)
211–310	3 (1.3)
311–410	0
411+	6 (2.6)
City of United Arab Emirates	
Sharjah	68 (30)
Dubai	36 (15.9)
Abu Dhabi	66 (29.1)
Ajman	38 (16.7)
Ras al Khaimah	8 (3.5)
Umm al Quwain	5 (2.2)
Fujairah	6 (2.6)

### Workplace features of participating pharmacists

Most of the community pharmacies are opened up to 12 h daily (78.4%). More than half of the participant (58.6%) claims that the average staff per shift is 2–5 staff. Additional information is displayed in Table 3.

**Table 3.** Work-related characteristics of the community pharmacists.

Study variables	Frequency (%)
Daily working hours of the pharmacy	
≤4	1 (0.4)
5–12	178 (78.4)
13–20	37 (16.3)
21+	11 (4.8)
Number of pharmacists during each shift	
≤1	67 (29.5)
2–5	133 (58.6)
6–9	14 (6.2)
10–13	6 (2.6)
14+	7 (3.1)

### Perception distribution among participants’ community pharmacists

There is a broad agreement among respondents (agree and strongly agree, 82.5%), regarding the necessity of PC services for all patients receiving medications and medication reviews (agree and strongly agree, 92.5%). The mainstream (92.5%) thinks that using pharmaceuticals to treat patients can lead to better results and 89.5% of the pharmacists agreed that they are responsible for identifying and resolving medicine-related problems. A significant majority (67.9%) agreed with the statement that continuing pharmacy education is not essential. More details are demonstrated in Table 4.

**Table 4.** Perception-elated questions and community pharmacists’ opinions on pharmaceutical care.

Questions	Frequency (%)					Median (IQR) scores
	Strongly disagree	Disagree	Not sure	Agree	Strongly agree	
1. The patients’ medications should be reviewed to prevent medicine-related errors and promote the appropriate use of medications	10 (4.4)	3 (1.3)	4 (1.8)	81 (35.7)	129 (56.8)	5 (4–5)
2. All patients receiving medicines require pharmaceutical care services	6 (2.6)	7 (3.1)	27 (11.9)	103 (45.5)	84 (37)	4 (4–5)
3. Pharmaceutical care can improve the patients’ treatment or health outcome	6 (2.6)	3 (1.3)	8 (3.5)	104 (45.8)	106 (46.7)	4 (4–5)
4. Pharmacists are professionally skilled health personnel in providing pharmaceutical care	6 (2.6)	3 (1.3)	11 (4.8)	92 (40.5)	115 (50.7)	5 (4–5)
5. Pharmacists are responsible for the identification, prevention and resolution of medicine-related problems	8 (3.5)	5 (2.2)	11 (4.8)	95 (41.9)	108 (47.6)	4 (4–5)
6. Continuing pharmacy education is NOT essential to equip pharmacists to provide pharmaceutical care*	18(7.9)	27 (11.9)	28 (12.3)	96 (42.39)	58 (25.6)	3.65 (3–5)

### Practice distribution among participants’ pharmacists

A total of 61.6% per cent of pharmacists said they usually or always review patient records. Also, (78.4%) of the participant pharmacists are counselling patients to prevent DTRPs and (58.5%) document patient information usually or all the time. A comprehensive approach to PC is reflected in the high percentage of pharmacists (70.9%), who usually or always consider the patient's overall conditions, while 82% are usually counselling patients on non-pharmacological approaches. The majority (74%) refer patients to doctors when necessary. Monitoring of adverse effects and treatment progress is a common practice among pharmacists surveyed (62.7% agreed and strongly agreed). More details are found in Table 5.

**Table 5.** Current practice-related questions and community pharmacists' opinions on pharmaceutical care.

Questions	Frequency (%)					Median (IQR) scores
	Never	Rare	Sometimes	Usually	All the time	
1. Enquiring about and reviewing patients’ medical and medicine records to decide if any intervention or recommendation must be made	8 (3.5)	16 (7)	63 (27.8)	75 (33)	65 (28.6)	4 (3–5)
2. Documenting patients’ clinical and medication information	13 (5.7)	25 (11)	56 (24.7)	53 (23.3)	80 (35.2)	4 (3–5)
3. Considering patients’ physical, socioeconomic and emotional conditions while providing PC*	7 (3.1)	16 (7.1)	43 (18.9)	92 (40.5)	69 (30.4)	4 (3–5)
4. Reviewing patients’ prescription or medication profile to determine possible DTRPs	3 (1.3)	11 (4.8)	48 (21.1)	81 (35.7)	84 (37)	4 (3–5)
5. Counselling patients to prevent potential DTRPs** and to promote the appropriate use of medicine	2 (0.9)	14 (6.2)	33 (14.5)	66 (29.1)	112 (49.3)	4 (4–5)
6. Resolving DTRPs of patients (e.g. Referring patient to a doctor or communicating with a doctor to resolve identified DTRPs)**	4 (1.8)	13 (5.7)	51 (22.5)	77 (33.9)	82 (36.1)	4 (4–5)
7. Counselling patient on non-pharmacological management of their illness	4 (1.8)	19 (8.4)	53 (23.3)	82 (36.1)	69 (30.4)	4 (3–5)
8. Referring patients to the doctor whenever necessary for further examination	1 (0.4)	9 (4)	49 (21.6)	76 (33.5)	92 (40.5)	4 (3–5)
9. Monitoring adverse effects of medicine	7 (3.1)	20 (8.8)	50 (22)	71 (31.3)	79 (34.8)	4 (3–5)
10. Monitoring patients’ treatment progress to ensure the achievement of therapeutic goal	8 (3.5)	19 (8.4)	60 (26.4)	69 (30.4)	71 (31.3)	4 (3–5)

*PC pharmaceutical care, IQR interquartile range

**DTRPs drug therapy-related problems (any unwanted incident related to medication therapy that actually or potentially affects the desired goals of treatment)

### Pharmaceutical care practices concerning the identification of drug therapy-related problems

Analysis of the data, regarding drug therapy-related problems encountered in a month, is demonstrated in Table 6. Dosing errors appear to be equally common, as indicated by the large majority of pharmacists (64.3%) who reported encountering between 1 and 100 errors in drug dose, frequency and duration. More details are shown in Table 6. Factors connected to obstacles encountered when administering pharmaceutical care by participating community pharmacists

**Table 6.** Community pharmacists’ practices toward identification of drug therapy-related problems in a month.

Study variables	Frequency (%)
Errors in drug dose, frequency and duration	
0	79 (34.8)
1–100	146 (64.3)
101–200	0
**201–300**	0
**301–400**	0
401+	2 (0.9)
Errors in drug name, dosage form and strength	
**0**	88 (38.8)
1–100	137 (60.4)
101–200	1 (0.4)
201–300	1 (0.4)
More than 300	0
Errors in drug-drug interaction	
0	92 (40.5)
1–100	134 (59)
**101–200**	0
201+	1 (0.4)
Errors in adverse drug reactions	
0	115 (50.7)
1+	112 (49.3)

Table 7 reveals significant concerns among PCs regarding barriers that they are facing in real life. Among notable results, pharmacists have serious concerns about the quality of training (43.6%) and guidelines (37.5%), the medical inter–professionals’ relations and cooperation (48.1% and 42.8%). The majority of respondents (64.3%) think that rather than providing comprehensive PC, the main goal of pharmacy practice is dispensing. Collectively, 81.5% per cent of participating pharmacists stated that the primary barrier was patients’ incapacity to understand prescription care instructions. It appears that the management of pharmacists is not in favour of the use of PC practices as 34.8% stated that there is no support from pharmacy owners. To a lesser degree, pharmacists believe that their communication skills (24.2%), documentation abilities (22.4%), attitude towards PC (16.7%) and self-confidence (18.5%) are seriously deficient. Jointly, nearly half of the participants agreed and strongly agreed about the lack of essential resources like private space for counselling (48.9%), time (45.4%), access to patient medical records (54.4%), reimbursement plan for provided services (44.1%) and computerised system that allow them to maintain the patients’ medical record (39.7%) or support them in medication assessment (36.6%).

**Table 7.** Community pharmacists' response to barrier-related questions.

Questions	Frequency (%)					Median (IQR) scores
	Strongly disagree	Disagree	Not sure	Agree	Strongly agree	
1. There is a lack of support from other health professionals towards pharmaceutical care	13 (5.7)	34 (15)	71 (31.3)	88 (38.8)	21 (9.3)	4 (3-5)
2. The co-ordination between pharmacists, doctors and other health professionals is poor	14 (6.2)	57 (25.1)	59 (26)	71 (31.3)	26 (11.5)	3 (2–4)
3. Patient is unable (due to illiteracy, unawareness or other reasons) to understand pharmaceutical care instructions	9 (4)	36 (15.9)	85 (37.4)	80 (35.2)	17 (7.5)	3 (3–4)
4. There is a lack of demand for and acceptance of pharmaceutical care by the patient	16 (7)	52 (22.9)	71 (31.3)	73 (32.2)	15 (6.6)	3 (2–4)
5. There is a lack of support from pharmacy owners or hospital administrators towards providing pharmaceutical care	17 (7.5)	72 (31.7)	59 (26)	60 (26.4)	19 (8.4)	3 (2–4)
6. There is a lack of supportive pharmaceutical care practice guideline	17 (7.5)	58 (25.6)	67 (29.5)	71 (31.3)	14 (6.2)	3 (2–4)
7. There is insufficient opportunity for pharmacists to interact closely with patients	13 (5.7)	64 (28.2)	62 (27.3)	71 (31.3)	17 (7.5)	3 (2–4)
8. Medicine practice and policy are more oriented towards medicine dispensing	10 (4.4)	17 (7.5)	54 (23.8)	119 (52.4)	27 (11.9)	4 (3–4)
9. Inadequate training is provided to pharmacists in providing pharmaceutical care	10 (4.4)	44 (19.4)	74 (32.6)	82 (36.1)	17 (7.5)	3 (3–4)
10. Pharmacists have inadequate therapeutic knowledge in resolving drug therapy-related problems	20 (8.8)	65 (28.6)	70 (30.8)	59 (26)	13 (5.7)	3 (2–4)
11. The education in the current pharmacy curriculum is inadequate to equip pharmacists to provide pharmaceutical care	24 (10.6)	56 (24.7)	67 (29.5)	70 (30.8)	10 (4.4)	3 (2–4)
12. Pharmacists lack skills in effective communication	29 (12.8)	93 (41)	50 (22)	44 (19.4)	11 (4.8)	2 (2–3)
13. Pharmacists lack skill in appropriate documentation	28 (12.3)	87 (38.3)	61 (26.9)	38 (16.7)	13 (5.7)	2 (2–3)
14. The attitude of pharmacists towards pharmaceutical care is inappropriate	31 (13.7)	102 (44.9)	56 (24.7)	30 (13.2)	8 (3.5)	2 (2–3)
15. Pharmacists lack self-confidence	44 (19.4)	93 (41)	48 (21.1)	33 (14.5)	9 (4)	2 (2–3)
16. Pharmacists lack motivation	38 (16.7)	73 (32.2)	40 (17.6)	49 (21.6)	27 (11.9)	3 (2–4)
17. There is a lack of compensation or reimbursement to pharmacists for providing pharmaceutical care	16 (7)	52 (22.5)	59 (26)	64 (28.2)	36 (15.9)	3 (2–4)
18. There is a lack of an appropriate computerised electronic system for maintaining the patients’ medical record	24 (10.6)	58 (25.6)	62 (27.3)	63 (27.8)	20 (8.8)	3 (2–4)
19. There is a lack of an appropriate computerised electronic system for medication assessment support	22 (9.7)	59 (26)	56 (24.7)	68 (30)	22 (9.7)	3 (2–4)
20. There is a lack of trained pharmacists to provide pharmaceutical care	20 (8.8)	66 (29.1)	57 (25.1)	68 (30)	16 (7)	3 (2–4)
21. There is insufficient pharmacist manpower	27 (11.9)	59 (26)	53 (23.3)	65 (28.6)	23 (10.1)	3 (2–4)
22. Pharmacists lack access to the patients’ medical record	22 (9.7)	53 (23.3)	54 (23.8)	78 (34.4)	20 (8.8)	3 (2–4)
23. There is insufficient time to provide pharmaceutical care	15 (6.6)	54 (23.8)	55 (24.2)	79 (34.8)	24 (10.6)	3 (2–4)
24. There is a lack of separate counselling areas for patients’ privacy	17 (7.5)	50 (22)	49 (21.6)	90 (39.6)	21 (9.3)	3 (2–4)
25. There is a lack of access to objective drug information sources	19 (8.4)	63 (27.8)	65 (28.6)	67 (29.5)	13 (5.7)	3 (2–4)

IQR interquartile range, DTRPs drug therapy-related problems

### Comparison of total perception, practice and barriers scores with demographic parameters

The total perception score was significant (*p* < 0.005) among four subgroups: age (*p* = 0.023), work experience (*p* = 0.036), current site of work (*p* = 0.025) and working hours (*p* = 0.012). The practice total score was significantly associated with work experience (*p* = 0.035) and training in pharmaceuticals (*p* = 0.009). The average number of pharmacists per shift was significantly associated with the total barriers score. PCs, who are alone in the shift (pharmacist per shift ≤1), had the highest correlation with the total barriers score (IQR = 83 (72–90)). The overall median (IQR) scores for perception, practice and barriers were 24 (22–26)/30, 40 (34–45)/50 and 76 (63–86)/125 respectively, Table 8.

**Table 8.** Total score of perceptions, practice and barriers to pharmaceutical care among subgroups of respondents.

Items	Perception score		Practice score		Barrier’s score	
	Total median score (IQR)	*p-*value	Total median score (IQR)	*p-*value	Total median score (IQR)	*p-*value
Gender						
Male	23.45 (22–26)	0.486	40.5 (36–45.75)	0.388	76.5 (64.25–89)	0.588
Female	24.97 (22–26)		39 (34–45)		76 (63–86)	
Age (in years)						
≤21	26 (23.5–28.5)	**0**.**023**	39 (33.5–50)	0.677	75 (29–94.5)	0.922
22–31	24.21 (23–26)		39.5 (34–45)		76 (63.75–85.25)	
32–41	22.72 (22–26)		34 (34.75–45)		75.5 (63.75–86)	
52+	22.16 (19.75–25.5)		37.5 (26.25–42.5)		76.5 (57.25–98.25)	
Qualification						
MPharm	24.58 (23–27)	0.346	40 (37–44)	0.718	76 (63–88)	0.096
PharmD	23.51 (22–27)		42 (35–46)		82 (70–97)	
BPharm	23.71 (22–26)		39 (33.5–45)		75 (62.5–85)	
Work experience (years)						
≤0	25 (24–27)	**0**.**036**	43 (39–48)	**0**.**035**	72 (38.5–83)	0.202
0.1–10.0	23.83 (22–26)		40 (34.5–45)		77 (65.5–86)	
10.1–20.0	23.26 (22–26)		37 (31.75–41.75)		74.5 (62.5–87.25)	
30.1+	21 (16)		41 (18)		56 (52)	
Training in PC						
Received	23.87 (23–26)	0.213	48 (36–46)	**0**.**009**	75.5 (63–85)	0.163
Not received	23.51 (22–26)		36 (32–43)		82 (68–89)	
The current site of work						
Community pharmacy (chain pharmacy)	24.02 (23–26)	**0**.**025**	39 (35–46)	0.533	76 (63–87)	0.829
Community pharmacy (independent pharmacy)	22.91 (22–26)		40 (34–43)		76 (66.75–85)	
Working hours						
≤34	22 (22–25)	**0**.**012**	39 (32.5–42.5)	0.123	75 (62.5–87.5)	0.694
35+	24.24 (22–26)		40 (35–46)		76 (63–86)	
Average number of pharmacists/shifts						
≤1	23.25 (22–26)	0.431	37 (32–42)	0.075	83 (72–90)	**0**.**002**
2–5	24.31 (22–26)		40 (35–45.5)		74 (59.5–84)	
6–9	23.78 (23–26.25)		41.5 (33–47)		64 (54–78.75)	
10–13	21 (16.75–26.25)		40.5 (33–47.75)		77 (61.5–93.5)	
14+	21.71 (22–27)		46 (36–49)		74 (65–80)	

*Significant *p-*values (*p* < 0.05) are in [bold]

### Post hoc analysis between paired groups

Post hoc analysis (Bonferroni test) of the statistically significant groups with more than three subgroups is mentioned in Table 9. The perception was significantly different between the two pairs of groups: more than 52 years vs. less than 21 years (*p* = 0.021) and 32–41 years vs. less than 21 years (*p* = 0.051). A significant difference in practice was seen between low experienced (less than or equal zero year) vs. 1–10 years (0.031) and low experienced (less than or equal zero year) vs. 10–20 years (0.004). More details can be seen in Table 9.

**Table 9.** Post hoc analysis results between paired groups.

Variables	Parameters	Paired groups	*P-*value
Perception	Age (in years)	More than 52 – less than 21	0.021
		32–41 vs. ≤21	0.015
	Work experience (years)	≥30 vs. ≥0	0.042
		1–10 vs. ≥0	0.032
Practice	Work experience (years)	10–20 vs. ≥0	0.004
		1–10 vs. ≥0	0.031
Barriers	Average number of pharmacists/shifts	6–9 vs. ≤1	0.002
		2–5 vs. ≤1	<0.001

### Correlation analysis of perception among perception-related questions and other variables

Kendall’s correlations were highly significant (*p* < 0.001) and corelated (tau ≥ 0.4) between C1 and C2, C1 and C3, C1 and C4, C1 and C5, C2 and C3, C2 and C4, C2 and C5, C3 and C4, C3 and C5, C4 and C5. Kendall’s correlations were highly significant but weakly correlated (tau = less than ±0.25) between C1 and working hours per week, C6 and experience, C3 with working hours per week and C4 with working hours per week. C6 was weekly corelated with experience (*r* = 0.224, *p* < 0.001), age (*r* = 0.170, *p* = 0.004) and daily prescriptions flow (*r* = 0.137, *p* = 0.019). More details are seen in Table 10 (Supplement: 1)

### Correlation analysis of practice among practice-related questions and other factors

Kendall’s correlations were highly significant (*p* value < 0.001) between All Construct (C1–C10), and also highly corelated between C1 and C2, C1 and C4, C1 and C5, C2 and C4, C2 and C5, C2 and C6, C2 and C10, C3 and C4, C3 and C5,C4 and C5, C4 and C6, C4 and C9, C4 and C10, C5 and C6, C5 and C7, C5 and C10 and finally between C9 and C10. Kendall’s correlations were highly significant but weakly correlated (tau = less than ±0.25) between others. Monitoring adverse effects was highly correlated with treatment progress (*r* = 0.627, *p* < 0.001). The same was seen between C4 and C5 (*r* = 0.615, *p* < 0.001). More data are seen in Table 11 (Supplement 2).

### Correlation among barrier-related various constructs and other barriers

Kendall’s correlations were moderate to strong but highly significant (*p-*value < 0.001) between most of the barrier’s constructs. A weak correlation but significant association (*p-*value < 0.05) was seen between qualification and C13 (*r* = −0.157, *p* = 0.007), C14 (*r* = −0.133, *p* = 0.025), C21 (*r* = −0.117, *p* = 0.043) and C24 (*r* = 0.134, *p* = 0.022). Same between experience and C1 (*r* = 0.121, *p* = 0.042) and C8 (*r* = 0.140, *p* = 0.02). The correlation was weak but significant between C9 and working hours (*r* = 0.121, *p* = 0.048) and between C11 and site of work (*r* = 0.133, *p* = 0.029). On the other hand, no significant correlation had been seen between age and barrier constructs. More data are seen in Table 12 (Supplement 3).

## Discussion

The present study findings show that CPs were generally good at trying to provide PC, and it seemed easy to get high enough. Belief in the benefits of providing PC was rated high indicating that pharmacists perceive benefits. However, their low rates of interactive PC actions suggest that they are facing multiple barriers and they don't have effective strategies to implement them (Farris & Schopflocher, 1999). This is the first quantitative, nationwide study that comprehensively examines CPs’ perception toward PC, the status of daily practice, and the barriers and their ability and desire to improve based on age, level of education and experience. More detailed views are explained in the below sections.

### Community pharmacists’ perception regarding pharmaceutical care

In this study, the participants’ pharmacists demonstrated a high perception of the PC concept. The median score of the 6 questions was highly towards the positive perception of PC. This finding is repetitive and consistent among PC studies worldwide, like in the USA (Christensen & Farris, 2006), Qatar (El Hajj et al., 2016) and Nigeria (Ma’aji & Ilyas, 2014). In this study, more specifically toward medication review, pharmacists expressed confidence that they are the qualified and responsible health professionals who can guarantee the best PC services.

Upon further analysis, findings revealed that PC perception scores are higher among younger, less experienced pharmacists. A direct reason could be because they are newly graduated pharmacists who have studied a more updated curriculum like pharmaceutical care, communication skills, patient-centred care, more focus on therapeutics, over-the-counter medications and extended experiential learning with more exposure to PC and latest evidence-based practice. Moreover, younger pharmacists may be more open to the evolving of new concepts and methods. This coincides with findings in other studies in Jordan (Jarab et al., 2024), Qatar (El Hajj et al., 2016), Iran (Mehralian et al., 2014), and UAE (Kharaba et al., 2022). Explaining the findings of older pharmacists with higher experience seemed to have lesser understanding, it might be because of multiple experiences they went through which might give them the feeling of having great practical experience, so they no more need the theoretical knowledge along with no exposure to updated pharmaceutical concepts and practices.

The survey results indicate that more than half of the participants agreed or strongly agreed that PC services are a basic right for all patients receiving medicine and that these services can improve treatment results. According to Ma’aji and Ilyas (2014), the majority of the participants had a positive attitude towards PC and considered it primarily a role and valuable practice that can improve patient health. This finding demonstrates that pharmacists are well aware of CP's important role in health care.

More than half of the participants believe that training and continuing education are necessary. Expansion of pharmacy education and training programmes was among the recommendations from Volmer et al. (2008), conducted in Estonia, Ngorsuraches and Li (2006), conducted in Thailand and Mohamed and Mahmoud (2016) in Sudan. CME (continuing medical education) is an obligatory requirement for the renewal of a pharmacist licence in the UAE. In UAE, this system can easily be integrated with PC practice by creating a special CME practical training system for pharmacists in order to be able to renew and maintain their licenses. This programne can be designed into levels that take the pharmacist from the basics of PC concepts to the higher level of PC professional practices, considering the level of experience for the pharmacists. In this way, both pharmacists and employers will comply. As a proactive step, integrating clinical practice and PC into the pharmacy curriculum will help ensure professional development and high-quality healthcare services in the future.

Finally, these findings indicate that most of the working CPs are agreeing towards the importance and essentiality of the PC concept and have a high desire to develop their skills and knowledge in PC.

### Practice of pharmaceutical care among community pharmacists

When it comes to the practical part, most observations in the literature discovered that pharmaceutical practice does not match with the high perception of almost all participants. Barely medication dispensing was seen among participants of the Alanazi et al. (2016) study.

In this study, a strong correlation between ‘Reviewing the patient’s prescription or medication profile to determine possible drug therapy-related problems or errors’ and ‘Counselling the patient to prevent potential drug-therapy-related problems and to promote appropriate use of medicine’ which indicate that PCs have strong adherence toward one of the main goals of PC concepts which are identifying and preventing drug-therapy related problem. On the other hand, the finding of Tang et al. (2020) was correlated with qualification as their findings showed that pharmacists with less education performed less effectively when reviewing prescriptions. The findings of this study show a sense of responsibility as pharmacy staff often consider them responsible for reviewing patients' prescriptions.

Another important finding related to the follow-up process which intended to evaluate the treatment progress of the patients, pharmacists in the study showed commitment to advise patients regarding proper ways for monitoring expected adverse effects or reactions. This finding is supported by a high correlation between counselling and resolving. Nevertheless, that was not consistent with studies conducted in Jordan (Jarab et al., 2024) and Qatar (El Hajj et al., 2016). further investigation is needed at this point as it raises a question regarding the awareness of PC toward adverse effects or reactions meanings and types. Their lack of interference in the treatment plan could be because of the lack of knowledge (McDonough et al., 1998).

As represented in this study, the majority of participants held a Bachelor of Pharmacy (BPharm) degree. While this is encouraging, it raises questions about professional and educational development opportunities in the field. It has been observed in some other studies like in Jordan (Jarab et al., 2024) that a higher educational level had contributed to a higher level of PC practice. In the Qatar study (El Hajj et al., 2016), most participants obtained their pharmacy degrees and had practised in their home country. The situation in Qatar mirrors in UAE, as the UAE is a multinational society and the labour market depends on expatriate workers from abroad. On one side, the difference in standards and educational methods leads to irregularities in the provision of PC. However, this situation provides a great set of experiences and knowledge that can be used to promote the best pharmaceutical practice.

In addition, most of the participants work full-time in chain pharmacies. This reflects that community pharmacy is a public practice in the UAE. Big chain community pharmacies represent most of the business model’s community pharmacies in the UAE market, which is why most of the participants had reported working in chain pharmacies. These two factors revealed a negative practice towards PC. The data reflects a busy work environment, where more than half of participants dispense between 11 and 110 prescriptions per day with less than 10 years of experience. This finding could be useful while standardising PC practical guidelines and policies for the plans. Chain pharmacies are the most pharmacies that accept fresh pharmacists with little experience. On the other side, they have well-structured internal processes and training programmes that usually concentrate on the business side rather than the pharmaceutical side. In Rayes et al. (2015) study, more than half of the participants claimed that pharmacy practice is turning into business-focused. It is possible to cooperate with these chains to accelerate pharmaceutical services and their implementation while maintaining the rate of profitability and business growth in general, but we need to apply pressure by-laws to ensure compliance.

Collectively, PC practice developed from the original definition of PC but has been applied in different contexts. It is always affected by many factors like cultural, economic and regulatory environments. PC services need to be aligned with the dynamic healthcare system and requirements of patients***.***

### Barriers encountered by community pharmacists in providing pharmaceutical care

In general, a high correlation between barriers questions reflects the high agreement of participants towards the mentioned barriers. Obstacles were similar and convergent between studies from different countries, for example, low acceptance from other providers and patients, lack of resources like time and space, inadequate compensation for pharmacists and absence of back from pharmacy proprietors (Ung et al., 2016). Ranking of barriers into major and minor was different between countries like in between Eldooma et al. (2023), Ma’aji and Ilyas (2014) and Amsler et al. (2001).

In the present study, the weak inter-professional support and cooperation along with the direction of recent policies were among the highest. Coincide findings were reflected from studies conducted in Poland (Kopciuch et al., 2021), and Malaysia (Loh et al., 2021).

Malaysian findings (Loh et al., 2021) said that more than two-third of their respondents had faced a lack of cooperation from medical prescribers as well as from patients themselves (Jones et al., 2005). Malaysian researchers categorised the ‘lack of supportive health care policies’ as one of the main barriers. Lack of awareness of social and interprofessional activities as well as effective regulatory policies may be among the main contributors to this situation. Communication with different medical specialities allows pharmacists to effectively contribute to prescription monitoring. A platform that enables them to view the details of the patient’s general prescription (prescription generator) may reduce the intensity of confrontation that may occur with other healthcare prescribers.

Most of the participants in this study claim that the recent direction of policies is more toward medication. The same result was shown in China, by Xi et al. (2019), as they found that pharmacists from chain pharmacies find the slow introduction of pharmacist law as a more relevant barrier. In the Ghazal (2014) study, 44% of the participants agreed that recent pharmacy management regulations are non-supportive, and change can’t occur without regulatory support in the UAE. The results of Rayes et al. (2015) from Dubai showed that 47.5% of the pharmacists in Dubai are facing a lack of legal protection against malpractice in their work. Such findings indicate a rapid concern about the need for laws and strong guidelines.

Looking into the high interquartile range (IQR) of total barriers related to pharmaceutical training indicated by a significant association between total practice score and pharmaceutical training, pharmacists who did not receive PC training are facing more obstacles. More than half of the Pharmacists in the KSA study, Siddique et al. (2022), well-defined PC same as those who considered their resource intensive or impractical. Pharmacists' worries about their ability to control their behaviour reflect a lack of practical implementation techniques (Farris & Schopflocher, 1999). Focusing on instructive programmes could help untrained CPs.

Lack of sufficient staff during the shift was one of the major barriers in most of the literature. As the *p-*value was highly significant, the majority of pharmacists who are alone in the shift claim that they feel unable to provide PC. The same finding in the study of Ibrahim and Scott (2013), Sallom et al. (2023) and Okonta et al. (2012). When pharmacists are alone, they may suffer from handling the workload, counselling patients and answering medication-related questions, leading to a compromised level of care. Unfortunately, due to the business view, most employers will hire only one staff per shift and forget or neglect the importance of providing PC with high efficiency which can’t be done with one staff only.

One observation from the data is that a majority of participants disagreed with the barriers that said pharmacists lack communication and documentation skills, attitudes toward PC, and self-confidence. This could highlight pharmacists' confidence in their expertise and their commitment to providing high-quality PC or could reflect the same finding of Rayes et al. (2015) from Dubai as they said it reflects the pharmacists' attempt to remove the blame from them or include them among the potential obstacles. It could also raise a question about their knowledge of the techniques of communication and proper documentation, actual vs. perceived providers (Rossing et al., 2005).

As discussed above, multiple barriers can limit the improvement of PC. The main barriers were medicine-oriented policies, lack of counselling area, lack of support and cooperation of other health care practitioners and insufficient time, and training. In addition, Kendall’s correlation was used to assess relationships between variables, and some results showed weak correlations. These correlations still offer valuable insights into patterns and future research and practice. Weak correlations will highlight areas where further investigations are needed. Furthermore, they can still guide hypothesis generation for further studies or suggest areas for targeted interventions.

## Limitations of the study

This study has several limitations. First, due to the random selection, the percentage of participants from each emirate such as Ras Al Khaimah and Fujairah may not be fully representative of the overall population which might have led to a certain level of sample bias. Second, the exclusion of pharmacy assistants and trainees from the study limits the generality of the findings, as they play a significant part in the pharmacy practice situation in UAE. Recall bias because the questionnaire was self-reported and a small sample size, both added restrictions to the generalisability of the results. The high percentage of middle-aged female pharmacists in the sample raises concerns about age and gender-related biases. These points highlight the need for care when extrapolating research findings to the general population of pharmacists and support the need for more detailed studies. Further, a low response rate of 61% can be a limitation though the achieved response rate is still reasonable given the study context and the busy nature of the targeted population. The lower response rate is attributed to the busy nature of the community pharmacies, some participants choose to withdraw because of workload (busy period) and time limit, also adding to the survey fatigue.

## Recommendations

Robust research studies are needed in collaboration with the three organisers of the healthcare sector which are the Ministry of Health (MOH), Dubai Health Authority (DHA) and Health Authority Abu Dhabi (HAAD). Development of comprehensive PC guidelines in collaboration with universities and researchers to ensure they are evidence-based (Castro & Correr, 2007) and to avoid dissonance between academic training and the actual needs of the CP practice. Also, PC reimbursement (in accordance with insurance parties) needs to be established to facilitate the PC implementation process (Eickhoff & Schulz, 2006).

Implementation of a one-year obligatory pre-graduation training programme would ensure that the new pharmacists are well-prepared to deliver effective PC.

Implementation of (a mystery patient programme) as a continuous way for the evaluation of PC services. Since the UAE community pharmacies sector is more toward business style, implementing of award of best PC provider may shift it towards more pharmaceutical style. Accessible national medical records platform and prescription generator as all prescriptions are generated from hospitals and clinics so PCs have low exposure to the prescription generation process (including GCC in future). Implementation of separate licenses, job descriptions and salary structures for PC providers.

## Conclusion

In this study, CPs across the UAE showed a positive perception toward PC. On the other hand, the level of practice was not on the same level of perception. Understandably, CPs are facing multiple barriers like weak interprofessional communications, the absence of policies and regulations, the dominance of profit-driven models and the lack of insurance reimbursement. Overall, enhancing the professionalism of CPs in the UAE requires substantial changes at multiple levels like stronger collaboration between academia and regulatory bodies, major restructuring of insurance and reimbursing policies, along with upgrading steps in licensing and training programmes. Additionally, increasing public awareness is also essential to improve patient acceptance of pharmacy services.

## Supplementary Material

Supplementary file 3.docx

Supplementary file 1.docx

Appendix 1.docx

Supplementary file 2.docx

## Data Availability

The datasets utilised and/or analysed in this study are available from the corresponding author upon reasonable request.
