# Supplementary material for: A national survey on community pharmacists’ perception, practice and perceived barriers towards pharmaceutical care services in the United Arab Emirates
Source: J Pharm Policy Pract. 2025 Jul 8;18(1):2523936. doi: 10.1080/20523211.2025.2523936 (PMC12239235; doi:10.1080/20523211.2025.2523936)
Supplement: Supplementary file 3.docx [file JPPP_A_2523936_SM9525.docx]

Table 12. The correlation among barrier-related constructs and other variables

| Questions /Variables | τ (p value) | | | | | | | | | | | | | | |  |
| --- | --- | --- | --- | --- | --- | --- | --- | --- | --- | --- | --- | --- | --- | --- | --- | --- |
|  | C1 | C2 | C3 | C4 | C5 | C6 | C7 | C8 | C9 | C10 | C11 | C12 | C13 | C14 | C15 | |
| C1 | - | 0.554  **(<0.001)** | 0.324  **(<0.001** | 0.378  **(<0.001)** | 0.510  **(<0.001)** | 0.499  **(<0.001)** | 0.358  **(<0.001)** | 0.366  **(<0.001)** | 0.421  **(<0.001)** | 0.301  **(<0.001)** | 0.368  **(<0.001)** | 0.195  **(<0.001)** | 0.223  **(<0.001)** | 0.242  **(<0.001)** | 0.201  **(<0.001)** | |
| C2 |  | - | 0.345  **(<0.001)** | 0.402  **(<0.001)** | 0.449  **(<0.001)** | 0.534  **(<0.001)** | 0.426  **(<0.001)** | 0.372  **(<0.001)** | 0.472  **(<0.001)** | 0.308  **(<0.001)** | 0.325  **(<0.001)** | 0.230  **(<0.001)** | 0.298  **(<0.001)** | 0.273  **(<0.001)** | 0.265  **(<0.001)** | |
| C3 |  |  | - | 0.513  **(<0.001)** | 0.357  **(<0.001)** | 0.322  **(<0.001)** | 0.373  **(<0.001)** | 0.353  **(<0.001)** | 0.249  **(<0.001)** | 0.130  **(0.02)** | 0.358  **(<0.001)** | 0.251  **(<0.001)** | 0.226  **(<0.001)** | 0.211  **(<0.001)** | 0.149  **(0.008)** | |
| C4 |  |  |  | - | 0.438  **(<0.001)** | 0.402  **(<0.001)** | 0.460  **(<0.001)** | 0.378  **(<0.001)** | 0.287  **(<0.001)** | 0.173  **(0.002)** | 0.359  **(<0.001)** | 0.283  **(<0.001)** | 0.232  **(<0.001)** | 0.266  **(<0.001)** | 0.208  **(<0.001)** | |
| C5 |  |  |  |  | - | 0.631  **(<0.001)** | 0.502  **(<0.001)** | 0.294  **(<0.001)** | 0.397  **(<0.001)** | 0.262  **(<0.001)** | 0.435  **(<0.001)** | 0.284  **(<0.001)** | 0.399  **(<0.001)** | 0.380  **(<0.001)** | 0.235  **(<0.001)** | |
| C6 |  |  |  |  |  | - | 0.513  **(<0.001)** | 0.260  **(<0.001)** | 0.535  **(<0.001)** | 0.321  **(<0.001)** | 0.430  **(<0.001)** | 0.296  **(<0.001)** | 0.345  **(<0.001)** | 0.357  **(<0.001)** | 0.289  **(<0.001)** | |
| C7 |  |  |  |  |  |  | - | 0.370  **(<0.001)** | 0.411  **(<0.001)** | 0.260  **(<0.001)** | 0.362  **(<0.001)** | 0.321  **(<0.001)** | 0.376  **(<0.001)** | 0.276  **(<0.001)** | 0.248  **(<0.001)** | |
| C8 |  |  |  |  |  |  |  | - | 0.359  **(<0.001)** | 0.222  **(<0.001)** | 0.283  **(<0.001)** | 0.213  **(<0.001)** | 0.298  **(<0.001)** | 0.234  **(<0.001)** | 0.208  **(<0.001)** | |
| C9 |  |  |  |  |  |  |  |  | - | 0.516  **(<0.001)** | 0.389  **(<0.001)** | 0.332  **(<0.001)** | 0.4  **(<0.001)** | 0.348  **(<0.001)** | 0.340  **(<0.001)** | |
| C10 |  |  |  |  |  |  |  |  |  | - | 0.385  **(<0.001)** | 0.343  **(<0.001)** | 0.364  **(<0.001)** | 0.363  **(<0.001)** | 0.337  **(<0.001)** | |
| C11 |  |  |  |  |  |  |  |  |  |  | - | 0.418  **(<0.001)** | 0.449  **(<0.001)** | 0.412  **(<0.001)** | 0.365  **(<0.001)** | |
| C12 |  |  |  |  |  |  |  |  |  |  |  | - | 0.653  **(<0.001)** | 0.552  **(<0.001)** | 0.553  **(<0.001)** | |
| C13 |  |  |  |  |  |  |  |  |  |  |  |  | - | 0.648  **(<0.001)** | 0.555  **(<0.001)** | |
| C14 |  |  |  |  |  |  |  |  |  |  |  |  |  | - | 0.576  **(<0.001)** | |
| C15 |  |  |  |  |  |  |  |  |  |  |  |  |  |  | - | |

| Questions /variables | τ (p value) | | | | | | | | | | | | | | | |
| --- | --- | --- | --- | --- | --- | --- | --- | --- | --- | --- | --- | --- | --- | --- | --- | --- |
|  | C16 | C17 | C18 | C19 | C20 | C21 | C22 | C23 | C24 | C25 | **Qualification** | **Experience (In years)** | **Site of work** | **Working hours per week** | **Age (in years)** | **Number of daily prescriptions handled** |
| C1 | 0.243  **(<0.001)** | 0.354  **(<0.001)** | 0.291  **(<0.001)** | 0.3  **(<0.001)** | 0.387  **(<0.001)** | 0.311  **(<0.001)** | 0.318  **(<0.001)** | 0.321  **(<0.001)** | 0.320  **(<0.001)** | 0.296  **(<0.001)** | 0.027  (0.653) | 0.121  **(0.042)** | 0.003  (0.960) | 0.114  (0.062) | 0.054  (0.364) | -0.001  (0.983) |
| C2 | 0.330  **(<0.001)** | 0.304  **(<0.001)** | 0.292  **(<0.001)** | 0.326  **(<0.001)** | 0.423  **(<0.001)** | 0.282  **(<0.001)** | 0.389  **(<0.001)** | 0.317  **(<0.001)** | 0.335  **(<0.001)** | 0.301  **(<0.001)** | 0.004  (0.939) | 0.093  (0.115) | 0.048  (0.424) | 0.01  (0.870) | -0.022  (0.709) | -0.105  (0.07) |
| C3 | 0.148  **(0.008)** | 0.215  **(<0.001)** | 0.180  (0.001) | 0.311  **(<0.001)** | 0.286  **(<0.001)** | 0.239  **(<0.001)** | 0.264  **(<0.001)** | 0.253  **(<0.001)** | 0.241  **(<0.001)** | 0.217  **(<0.001)** | -0.073  (0.218) | 0.03  (0.614) | 0.109  (0.077) | -0.003  (0.963) | 0.061  (0.308) | -0.069  (0.246) |
| C4 | 0.212  **(<0.001)** | 0.255  **(<0.001)** | 0.249  **(<0.001)** | 0.301  **(<0.001)** | 0.313  **(<0.001)** | 0.296  **(<0.001)** | 0.256  **(<0.001)** | 0.303  **(<0.001)** | 0.290  **(<0.001)** | 0.267  **(<0.001)** | 0.022  (0.702) | 0.035  (0.553) | 0.054  (0.378) | 0  (0.995) | -0.025  (0.669) | -0.012  (0.841) |
| C5 | 0.306  **(<0.001)** | 0.407  **(<0.001)** | 0.434  **(<0.001)** | 0.483  **(<0.001)** | 0.472  **(<0.001)** | 0.385  **(<0.001)** | 0.450  **(<0.001)** | 0.399  **(<0.001)** | 0.410  **(<0.001)** | 0.392  **(<0.001)** | -0.08  (0.760) | 0.027  (0.644) | -0.004  (0.942) | -0.003  (0.958) | -0.031  (0.601) | 0.019  (0.739) |
| C6 | 0.337  **(<0.001)** | 0.441  **(<0.001)** | 0.425  **(<0.001)** | 0.449  **(<0.001)** | 0.445  **(<0.001)** | 0.362  **(<0.001)** | 0.457  **(<0.001)** | 0.355  **(<0.001)** | 0.364  **(<0.001)** | 0.398  **(<0.001)** | 0.006  (0.920) | 0.034  (0.567) | 0.012  (0.849) | 0.021  (0.732) | 0.029  (0.622) | -0.077  (0.187) |
| C7 | 0.274  **(<0.001)** | 0.368  **(<0.001)** | 0.405  **(<0.001)** | 0.420  **(<0.001)** | 0.397  **(<0.001)** | 0.384  **(<0.001)** | 0.385  **(<0.001)** | 0.4  **(<0.001)** | 0.371  **(<0.001)** | 0.326  **(<0.001)** | 0.073  (0.217) | 0.029  (0.638) | 0.064  (0.289) | -0.01  (0.872) | 0.068  (0.251) | -0.059  (0.311) |
| C8 | 0.256  **(<0.001)** | 0.260  **(<0.001)** | 0.223  **(<0.001)** | 0.271  **(<0.001)** | 0.258  **(<0.001)** | 0.243  **(<0.001)** | 0.253  **(<0.001)** | 0.298  **(<0.001)** | 0.319  **(<0.001)** | 0.201  **(<0.001)** | -0.049  (0.415) | 0.140  **(0.02)** | -0.061  (0.323) | 0.084  (0.175) | 0.06  (0.321) | 0.015  (0.802) |
| C9 | 0.3  **(<0.001)** | 0.377  **(<0.001)** | 0.280  **(<0.001)** | 0.290  **(<0.001)** | 0.408  **(<0.001)** | 0.282  **(<0.001)** | 0.309  **(<0.001)** | 0.351  **(<0.001)** | 0.360  **(<0.001)** | 0.367  **(<0.001)** | -0.051  (0.390) | 0.005  (0.929) | -0.017  (0.786) | 0.121  **(0.048)** | 0.067  (0.262) | -0.006  (0.926) |
| C10 | 0.239  **(<0.001)** | 0.167  **(0.002)** | 0.126  **(0.022)** | 0.149  **(0.007)** | 0.283  **(<0.001)** | 0.205  **(<0.001)** | 0.133  **(0.016)** | 0.264  **(<0.001)** | 0.156  **(0.005)** | 0.245  **(<0.001)** | -0.06  (0.307) | 0  (0.998) | -0.049  (0.420) | 0.049  (0.422) | -0.018  (0.763) | -0.009  (0.872) |
| C11 | 0.222  **(<0.001)** | 0.267  **(<0.001)** | 0.231  **(<0.001)** | 0.226  **(<0.001)** | 0.385  **(<0.001)** | 0.251  **(<0.001)** | 0.269  **(<0.001)** | 0.272  **(<0.001)** | 0.356  **(<0.001)** | 0.448  **(<0.001)** | -0.027  (0.647) | -0.01  (0.861) | 0.133  **(0.029)** | -0.069  (0.254) | -0.003  (0.964) | -0.073  (0.213) |
| C12 | 0.361  **(<0.001)** | 0.235  **(<0.001)** | 0.199  **(<0.001)** | 0.241  **(<0.001)** | 0.414  **(<0.001)** | 0.283  **(<0.001)** | 0.236  **(<0.001)** | 0.250  **(<0.001)** | 0.254  **(<0.001)** | 0.354  **(<0.001)** | -0.091  (0.122) | 0.046  (0.433) | 0.024  90.687) | -0.023  (0.703) | 0.089  (0.133) | -0.061  (0.3) |
| C13 | 0.436  **(<0.001)** | 0.366  **(<0.001)** | 0.363  **(<0.001)** | 0.368  **(<0.001)** | 0.467  **(<0.001)** | 0.311  **(<0.001)** | 0.384  **(<0.001)** | 0.325  **(<0.001)** | 0.368  **(<0.001)** | 0.368  **(<0.001)** | -0.157  **(0.007)** | 0.037  (0.534) | 0.008  (0.891) | -0.058  (0.340) | 0.085  (0.149) | -0.110  **(0.06)** |
| C14 | 0.433  **(<0.001)** | 0.330  **(<0.001)** | 0.314  **(<0.001)** | 0.373  **(<0.001)** | 0.403  **(<0.001)** | 0.267  **(<0.001)** | 0.333  **(<0.001)** | 0.311  **(<0.001)** | 0.306  **(<0.001)** | 0.365  **(<0.001)** | -0.133  **(0.025)** | -0.013  (0.832) | -0.035  (0.566) | -0.017  (0.778) | 0.015  (0.8) | -0.136  **(0.021)** |
| C15 | 0.564  **(<0.001)** | 0.3  **(<0.001)** | 0.254  **(<0.001)** | 0.282  **(<0.001)** | 0.367  **(<0.001)** | 0.301  **(<0.001)** | 0.260  **(<0.001)** | 0.270  **(<0.001)** | 0.252  **(<0.001)** | 0.330  **(<0.001)** | -0.052  (0.380) | 0.027  (0.649) | -0.018  (0.768) | -0.036  (0.559) | 0.076  (0.2) | -0.112  (0.057) |

| Questions /variables | τ (p value) | | | | | | | | | | | | | | |
| --- | --- | --- | --- | --- | --- | --- | --- | --- | --- | --- | --- | --- | --- | --- | --- |
|  | C17 | C18 | C19 | C20 | C21 | C22 | C23 | C24 | C25 | **Qualification** | **Experience (In years)** | **Site of work** | **Working hours per week** | **Age (in years)** | **Number of daily prescriptions handled** |
| C16 | 0.502  **(<0.001)** | 0.359  **(<0.001)** | 0.387  **(<0.001)** | 0.4  **(<0.001)** | 0.396  **(<0.001)** | 0.406  **(<0.001)** | 0.427  **(<0.001)** | 0.387  **(<0.001)** | 0.281  **(<0.001)** | -0.061  (0.294) | 0.036  (0.540) | 0.076  (0.206) | -0.003  (0.956) | 0.061  (0.295) | -0.097  (0.095) |
| C17 | - | 0.512  **(<0.001)** | 0.517  **(<0.001)** | 0.387  **(<0.001)** | 0.441  **(<0.001)** | 0.5  **(<0.001)** | 0.539  **(<0.001)** | 0.507  **(<0.001)** | 0.368  **(<0.001)** | -0.108  (0.062) | 0.028  (0.629) | 0.021  (0.721) | 0.1  (0.095) | 0.011  (0.846) | -0.059  (0.304) |
| C18 |  | - | 0.707  **(<0.001)** | 0.398  **(<0.001)** | 0.424  **(<0.001)** | 0.543  **(<0.001)** | 0.411  **(<0.001)** | 0.474  **(<0.001)** | 0.388  **(<0.001)** | -0.021  (0.723) | 0.091  (0.751) | 0.006  (0.924) | -0.009  (0.884) | 0.043  (0.465) | -0.048  (0.404) |
| C19 |  |  | - | 0.427  **(<0.001)** | 0.471  **(<0.001)** | 0.556  **(<0.001)** | 0.520  **(<0.001)** | 0.494  **(<0.001)** | 0.419  **(<0.001)** | -0.107  (0.065) | 0.02  (0.730) | -0.044  (0.465) | 0.045  (0.454) | 0.021  (0.715) | -0.007  (0.899) |
| C20 |  |  |  | - | 0.473  **(<0.001)** | 0.454  **(<0.001)** | 0.454  **(<0.001)** | 0.460  **(<0.001)** | 0.44  **(<0.001)** | -0.096  (0.102) | 0.092  (0.117) | 0.052  (0.388) | -0.022  (0.719) | 0.095  (0.108) | -0.055  (0.346) |
| C21 |  |  |  |  | - | 0.439  **(<0.001)** | 0.543  **(<0.001)** | 0.458  **(<0.001)** | 0.392  **(<0.001)** | -0.117  **(0.043)** | 0.062  (0.291) | 0.051  (0.397) | -0.028  (0.640) | -0.03  (0.618) | 0.067  (0.250) |
| C22 |  |  |  |  |  | - | 0.5  **(<0.001)** | 0.523  **(<0.001)** | 0.460  **(<0.001)** | -0.120  0.04) | 0.004  (0.949) | 0.041  (0.495) | 0.041  (0.495) | -0.013  (0.826) | -0.125  **(0.032)** |
| C23 |  |  |  |  |  |  | **-** | 0.572  **(<0.001)** | 0.426  **(<0.001)** | 0.133  (0.023) | 0.041  (0.488**)** | 0.033  (0.590) | 0.08  (0.188) | -0.021  (0.727) | 0.006  (0.921) |
| C24 |  |  |  |  |  |  |  | - | 0.524  **(<0.001)** | -0.134  **(0.022)** | 0.065  (0.269) | 0.038  (0.534) | 0.1  (0.101) | 0.003  (0.954) | -0.035  (0.555) |
| C25 |  |  |  |  |  |  |  |  | - | -0.071  (0.228) | -0.002  (0.970) | 0.039  (0.521) | 0.081  (0.181) | -0.03  (0.607) | -0.065  (0.266) |
| Qualification |  |  |  |  |  |  |  |  |  | - | # | **#** | # | # | # |
| Experience (in years) |  |  |  |  |  |  |  |  |  |  | - | # | # | # | # |
| Site of work |  |  |  |  |  |  |  |  |  |  |  | - | # | # | **#** |
| Working hours per week |  |  |  |  |  |  |  |  |  |  |  |  | - | # | **#** |
| Number of daily prescriptions handled |  |  |  |  |  |  |  |  |  |  |  |  |  | - | # |

C1: There is a lack of support from other health professionals toward pharmaceutical care.

C2: The co-ordination between pharmacists, doctors and other health professionals is poor.

C3: Patient is unable (due to illiteracy, unawareness, or other reasons) to understand pharmaceutical care instructions.

C4: There is a lack of demand for and acceptance of pharmaceutical care by the patient.

C5: There is a lack of support from pharmacy owners or hospital administrators toward providing pharmaceutical care.

C6: There is a lack of supportive pharmaceutical care practice guideline.

C7: There is insufficient opportunity for pharmacists to interact closely with patients.

C8: Medicine practice and policy are more oriented toward medicine dispensing.

C9: Inadequate training is provided to pharmacist in providing pharmaceutical care.

C10: Pharmacists have inadequate therapeutic knowledge in resolving drug therapy-related problems.

C11: The education in the current pharmacy curriculum is inadequate to equip pharmacists to provide pharmaceutical care.

C12: Pharmacists lack skill in effective communication.

C13: Pharmacists lack skill in appropriate documentation.

C14: The attitude of pharmacists toward pharmaceutical care is inappropriate.

C15: Pharmacists lack self-confidence.

C16: Pharmacists lack motivation.

C17: There is lack of compensation or reimbursement to pharmacists for providing pharmaceutical care.

C18: There is a lack of appropriate computerized electronic system for maintaining the patients’ medical record.

C19: There is a lack of appropriate computerized electronic system for medication assessment support.

C20: There is a lack of trained pharmacist to provide pharmaceutical care.

C21: There is insufficient pharmacist manpower.

C22: Pharmacists lack access to the patient medical record.

C23: There is insufficient time to provide pharmaceutical care.

C24: There is lack of separate counselling area for patient’s privacy.

C25: There is lack of access to objective drug information sources.

C: Construct; τ: Kendall’s correlation (Tau); bold p value < 0.001
