# Supplementary material for: A national survey on community pharmacists’ perception, practice and perceived barriers towards pharmaceutical care services in the United Arab Emirates
Source: J Pharm Policy Pract. 2025 Jul 8;18(1):2523936. doi: 10.1080/20523211.2025.2523936 (PMC12239235; doi:10.1080/20523211.2025.2523936)
Supplement: Supplementary file 1.docx [file JPPP_A_2523936_SM9524.docx]

**Supplementary:**

Supplementary 1: Table 10 The correlation among perception-related questions and other variables

| Questions /variables | τ (p value) | | | | | | | | | | | |
| --- | --- | --- | --- | --- | --- | --- | --- | --- | --- | --- | --- | --- |
|  | **C1** | **C2** | **C3** | **C4** | **C5** | **C6** | **Qualification** | **Experience (In years)** | **Site of work** | **Working hours per week** | **Age (in years)** | **Number of daily prescriptions handled** |
| C1 | - | **0.5**  **(<0.001)** | **0.453**  **(<0.001)** | **0.518**  **(<0.001)** | **0.501**  **(<0.001)** | 0.141  **(0.017)** | -0.127  **(0.042)** | -0.025  **(0.017)** | -0.125  (0.053) | 0.249  **(<0.001**) | -0.089  (0.157) | 0.065  (0.298) |
| C2 |  | - | **0.589**  **(<0.001)** | **0.539**  **(<0.001)** | **0.5**  **(<0.001)** | 0.089  (0.119) | -0.062  (0.303) | -0.113  (0.065) | -0.057  (0.362) | 0.138  **(0.027)** | -0.102  (0.096) | 0.028  (0.639) |
| C3 |  |  | - | **0.553**  **(<0.001)** | **0.585**  **(<0.001)** | 0.176  **(0.003)** | -0.050  (0.425) | -0.078  (0.212) | -0.149  **(0.021)** | 0.216  **(<0.001)** | -0.139  **(0.027)** | 0.064  (0.305) |
| C4 |  |  |  | - | **0.599**  **(<0.001)** | 0.104  (0.077) | -0.033  (0.592) | -0.025  (0.691) | -0.136  **(0.033)** | 0.209  **(<0.001)** | -0.089  (0.155) | 0.131  **(0.035)** |
| C5 |  |  |  |  | - | 0.185  **(0.001)** | -0.053  (0.394) | -0.008  (0.9) | -0.117  (0.065) | 0.158  **(0.013)** | -0.061  (0.329) | 0.143  **(0.02)** |
| C6 |  |  |  |  |  | - | 0.006  (0.925) | 0.224  **(<0.001)** | 0.009  (0.881) | 0.117  (0.054) | 0.170  **(0.004)** | 0.137  **(0.019)** |
| Qualification |  |  |  |  |  |  | - | 0.030  (0.635) | -0.097  (0.133) | -0.016  (0.802) | -0.102  (0.103) | -0.010  (0.872) |
| Experience (in years) |  |  |  |  |  |  |  | - | 0.062  (0.339) | 0.175  **(0.007)** | **0.567**  **(<0.001)** | 0.108  (0.083) |
| Site of work |  |  |  |  |  |  |  |  | - | -0.107  (0.109) | 0.082  (0.205) | 0.025  (0.701) |
| Working hours per week |  |  |  |  |  |  |  |  |  | - | 0.130  **(0.045)** | 0.119  (0.063) |
| Age (in years) |  |  |  |  |  |  |  |  |  |  | - | 0.041  (0.515) |

C1: Patient’s medications should be reviewed to prevent medicine-related errors and promote appropriate use of medications.

C2: All patients receiving medicines require PC.

C3: PC can improve patient’s treatment or health outcome.

C4: Pharmacists are professionally skilled HPs in providing PC.

C5: Pharmacists are responsible for the identification, prevention, and resolution of MRPs.

C6: Continuing pharmacy education is NOT essential to equip pharmacists to provide PC.

C: Construct; τ: Kendall’s correlation (Tau).

**p value < 0.001
