# Supplementary material for: A national survey on community pharmacists’ perception, practice and perceived barriers towards pharmaceutical care services in the United Arab Emirates
Source: J Pharm Policy Pract. 2025 Jul 8;18(1):2523936. doi: 10.1080/20523211.2025.2523936 (PMC12239235; doi:10.1080/20523211.2025.2523936)
Supplement: Appendix 1.docx [file JPPP_A_2523936_SM9523.docx]

**Appendix 1**

**The study questionnaire**

**Demographic and work-related information**

**1- Age:**

○ ≤ 21 ○ 22–31 ○ 32–41 ○ 52+

**2- Gender**

○ Male ○ Female

**3- Qualification**

○ MPharm ○ PharmD ○ BPharm

**4- Work experiences (in years)**

○ ≤ 0 ○ 0.1–10.0 ○ 10.1–20.0 ○ 30.1+

**5- Training in pharmaceutical care**

○ Received ○ Not received

**6- Current site of work**

○ Community pharmacy (chain pharmacy) ○ Community pharmacy (independent pharmacy)

**7- Working hours per week**

○ ≤ 34 ○ 35+

**8- Daily number of prescriptions handled**

○ ≤ 10 ○ 11–110 ○ 111–210 ○ 211–310 ○ 411+

**9- City of UAE**

○ Sharjah ○ Dubai ○ Abu Dhabi ○ Ajman ○ Ras Al-Khaimah ○ Umm AL Quwain ○ Fujairah

**10- Daily working hours of pharmacy**

○ ≤ 4 ○ 5–12 ○ 13–20 ○ 21+

**11- Number of pharmacists during each shift**

○ ≤ 1 ○ 2–5 ○ 6–9 ○ 10–13 ○ 14+

**Perception (current level of knowledge)-related questions**

| **Questions** | **Strongly disagree** | **Disagree** | **Not sure** | **Agree** | **Strongly agree** |
| --- | --- | --- | --- | --- | --- |
| **1- Patient’s medications should be reviewed to prevent medicine-related errors and promote appropriate use of medications** |  |  |  |  |  |
| **2- All patients receiving medicines require pharmaceutical care services** |  |  |  |  |  |
| **3- Pharmaceutical care can improve patient’s treatment or health outcome** |  |  |  |  |  |
| **4- Pharmacists are professionally skilled health personnel in providing pharmaceutical care** |  |  |  |  |  |
| **5- Pharmacists are responsible for identification, prevention and resolution of medicine-related problems** |  |  |  |  |  |
| **6- Continuing pharmacy education is NOT essential to equip pharmacists to provide pharmaceutical care** |  |  |  |  |  |

**Practice (attitude)-related questions**

| **Questions** | **Never** | **Rare** | **Sometimes** | **Usually,** | **All the time** |
| --- | --- | --- | --- | --- | --- |
| **1- Enquiring about and reviewing patient’s medical and medicine records to decide if any intervention or recommendation must be made** |  |  |  |  |  |
| **2- Documenting patient’s clinical and medication information** |  |  |  |  |  |
| **3- Considering patient’s physical, socioeconomic, and emotional conditions while providing PC** |  |  |  |  |  |
| **4- Reviewing patient’s prescription or medication profile to determine possible DTRPs** |  |  |  |  |  |
| **5- Counselling patient to prevent potential DTRPs and to promote appropriate use of medicine** |  |  |  |  |  |
| **6- Resolving DTRPs of patient (e.g., Referring patient to doctor or communicating with doctor to resolve identified DTRPs)** |  |  |  |  |  |
| **7- Counselling patient on non-pharmacological management of their illness** |  |  |  |  |  |
| **8- Referring patients to doctor whenever necessary for further examination** |  |  |  |  |  |
| **9- Monitoring adverse effects of medicine** |  |  |  |  |  |
| **10- Monitoring patient’s treatment progress to assure achievement of therapeutic goal** |  |  |  |  |  |

**DTRP identification-related questions**

**1- Errors on drug dose, frequency, and duration**

○ 0 ○ 1–100 ○ 101–200 ○ 401+

**2- Errors on drug name, dosage form and strength**

○ 0 ○ 1–100 ○ 101–200 ○ 201–300 ○ 501+

**3- Errors on drug-drug interaction**

○ 0 ○ 1–100 ○ 201+

**4- Errors on adverse drug reactions**

○ 0 ○ 1+

**Barrier-related questions**

| **Questions** | **Strongly disagree** | **Disagree** | **Not sure** | **Agree** | **Strongly agree** |
| --- | --- | --- | --- | --- | --- |
| **1- There is a lack of support from other health professionals toward pharmaceutical care** |  |  |  |  |  |
| **2- The co-ordination between pharmacists, doctors and other health professionals is poor** |  |  |  |  |  |
| **3- Patient is unable (due to illiteracy, unawareness or other reasons) to understand pharmaceutical care instructions** |  |  |  |  |  |
| **4- There is a lack of demand for and acceptance of pharmaceutical care by the patient** |  |  |  |  |  |
| **5- There is a lack of support from pharmacy owners or hospital administrators toward providing pharmaceutical care** |  |  |  |  |  |
| **6- There is a lack of supportive pharmaceutical care practice guideline** |  |  |  |  |  |
| **7- There is insufficient opportunity for pharmacists to interact closely with patients** |  |  |  |  |  |
| **8- Medicine practice and policy are more oriented toward medicine dispensing** |  |  |  |  |  |
| **9- Inadequate training is provided to pharmacist in providing pharmaceutical care** |  |  |  |  |  |
| **10- Pharmacists have inadequate therapeutic knowledge in resolving drug therapy-related problems** |  |  |  |  |  |
| **11- The education in the current pharmacy curriculum is inadequate to equip pharmacists to provide pharmaceutical care** |  |  |  |  |  |
| **12- Pharmacists lack skill in effective communication** |  |  |  |  |  |
| **13- Pharmacists lack skill in appropriate documentation** |  |  |  |  |  |
| **14- The attitude of pharmacists toward pharmaceutical care is inappropriate** |  |  |  |  |  |
| **15- Pharmacists lack self-confidence** |  |  |  |  |  |
| **16- Pharmacists lack motivation** |  |  |  |  |  |
| **17- There is lack of compensation or reimbursement to pharmacists for providing pharmaceutical care** |  |  |  |  |  |
| **18- There is a lack of appropriate computerized electronic system for maintaining the patients’ medical record** |  |  |  |  |  |
| **19- There is a lack of appropriate computerized electronic system for medication assessment support** |  |  |  |  |  |
| **20- There is a lack of trained pharmacist to provide pharmaceutical care** |  |  |  |  |  |
| **21- There is insufficient pharmacist manpower** |  |  |  |  |  |
| **22- Pharmacists lack access to the patient medical record** |  |  |  |  |  |
| **23- There is insufficient time to provide pharmaceutical care** |  |  |  |  |  |
| **24- There is lack of separate counselling area for patient’s privacy** |  |  |  |  |  |
| **25- There is lack of access to objective drug information sources** |  |  |  |  |  |
