# Supplementary material for: A national survey on community pharmacists’ perception, practice and perceived barriers towards pharmaceutical care services in the United Arab Emirates
Source: J Pharm Policy Pract. 2025 Jul 8;18(1):2523936. doi: 10.1080/20523211.2025.2523936 (PMC12239235; doi:10.1080/20523211.2025.2523936)
Supplement: Supplementary file 2.docx [file JPPP_A_2523936_SM9522.docx]

Table. 11. The correlation among practice-related questions and other variables

| Questions /Variables | τ (p value) | | | | | | | | | | | | | | |  |
| --- | --- | --- | --- | --- | --- | --- | --- | --- | --- | --- | --- | --- | --- | --- | --- | --- |
|  | C1 | C2 | C3 | C4 | C5 | C6 | C7 | C8 | C9 | C10 | C11 | C12 | C13 | C14 | C15 | |
| C1 | - | 0.554  **(<0.001)** | 0.324  **(<0.001** | 0.378  **(<0.001)** | 0.510  **(<0.001)** | 0.499  **(<0.001)** | 0.358  **(<0.001)** | 0.366  **(<0.001)** | 0.421  **(<0.001)** | 0.301  **(<0.001)** | 0.368  **(<0.001)** | 0.195  **(<0.001)** | 0.223  **(<0.001)** | 0.242  **(<0.001)** | 0.201  **(<0.001)** | |
| C2 |  | - | 0.345  **(<0.001)** | 0.402  **(<0.001)** | 0.449  **(<0.001)** | 0.534  **(<0.001)** | 0.426  **(<0.001)** | 0.372  **(<0.001)** | 0.472  **(<0.001)** | 0.308  **(<0.001)** | 0.325  **(<0.001)** | 0.230  **(<0.001)** | 0.298  **(<0.001)** | 0.273  **(<0.001)** | 0.265  **(<0.001)** | |
| C3 |  |  | - | 0.513  **(<0.001)** | 0.357  **(<0.001)** | 0.322  **(<0.001)** | 0.373  **(<0.001)** | 0.353  **(<0.001)** | 0.249  **(<0.001)** | 0.130  **(0.02)** | 0.358  **(<0.001)** | 0.251  **(<0.001)** | 0.226  **(<0.001)** | 0.211  **(<0.001)** | 0.149  **(0.008)** | |
| C4 |  |  |  | - | 0.438  **(<0.001)** | 0.402  **(<0.001)** | 0.460  **(<0.001)** | 0.378  **(<0.001)** | 0.287  **(<0.001)** | 0.173  **(0.002)** | 0.359  **(<0.001)** | 0.283  **(<0.001)** | 0.232  **(<0.001)** | 0.266  **(<0.001)** | 0.208  **(<0.001)** | |
| C5 |  |  |  |  | - | 0.631  **(<0.001)** | 0.502  **(<0.001)** | 0.294  **(<0.001)** | 0.397  **(<0.001)** | 0.262  **(<0.001)** | 0.435  **(<0.001)** | 0.284  **(<0.001)** | 0.399  **(<0.001)** | 0.380  **(<0.001)** | 0.235  **(<0.001)** | |
| C6 |  |  |  |  |  | - | 0.513  **(<0.001)** | 0.260  **(<0.001)** | 0.535  **(<0.001)** | 0.321  **(<0.001)** | 0.430  **(<0.001)** | 0.296  **(<0.001)** | 0.345  **(<0.001)** | 0.357  **(<0.001)** | 0.289  **(<0.001)** | |
| C7 |  |  |  |  |  |  | - | 0.370  **(<0.001)** | 0.411  **(<0.001)** | 0.260  **(<0.001)** | 0.362  **(<0.001)** | 0.321  **(<0.001)** | 0.376  **(<0.001)** | 0.276  **(<0.001)** | 0.248  **(<0.001)** | |
| C8 |  |  |  |  |  |  |  | - | 0.359  **(<0.001)** | 0.222  **(<0.001)** | 0.283  **(<0.001)** | 0.213  **(<0.001)** | 0.298  **(<0.001)** | 0.234  **(<0.001)** | 0.208  **(<0.001)** | |
| C9 |  |  |  |  |  |  |  |  | - | 0.516  **(<0.001)** | 0.389  **(<0.001)** | 0.332  **(<0.001)** | 0.4  **(<0.001)** | 0.348  **(<0.001)** | 0.340  **(<0.001)** | |
| C10 |  |  |  |  |  |  |  |  |  | - | 0.385  **(<0.001)** | 0.343  **(<0.001)** | 0.364  **(<0.001)** | 0.363  **(<0.001)** | 0.337  **(<0.001)** | |
| C11 |  |  |  |  |  |  |  |  |  |  | - | 0.418  **(<0.001)** | 0.449  **(<0.001)** | 0.412  **(<0.001)** | 0.365  **(<0.001)** | |
| C12 |  |  |  |  |  |  |  |  |  |  |  | - | 0.653  **(<0.001)** | 0.552  **(<0.001)** | 0.553  **(<0.001)** | |
| C13 |  |  |  |  |  |  |  |  |  |  |  |  | - | 0.648  **(<0.001)** | 0.555  **(<0.001)** | |
| C14 |  |  |  |  |  |  |  |  |  |  |  |  |  | - | 0.576  **(<0.001)** | |
| C15 |  |  |  |  |  |  |  |  |  |  |  |  |  |  | - | |

C1: Enquiring about and reviewing patient’s medical and medicine records to decide if any intervention or recommendation must be made.

C2: Documenting patient’s clinical and medication information record.

C3: Considering patient’s conditions (physical, social, emotional, economic etc.) while providing pharmaceutical care.

C4: Reviewing the patient’s prescription or medication profile to determine possible drug therapy-related problems or errors.

C5: Counselling the patient to prevent potential drug-therapy related problem and to promote appropriate use of medicine.

C6: Resolving the drug therapy-related problem of patient. (e.g., referring the patient to doctor or communicating with the doctor to resolve the identified drug therapy-related problem).

C7: Counselling the patient on non-pharmacological management of their illness.

C8: Referring patients to doctor whenever necessary for further examination.

C9: Monitoring adverse effects or reactions of medicine in patient.

C10: Monitoring patient’s treatment progress to assure the achievement of therapeutic goal.

C: Construct; τ: Kendall’s correlation (Tau); Bold p value < 0.001.
